# Systems Radiology and Personalized Medicine

**DOI:** 10.3390/jpm11080769

**Published:** 2021-08-04

**Authors:** Wouter Foppen, Nelleke Tolboom, Pim A. de Jong

**Affiliations:** Department of Radiology and Nuclear Medicine, University Medical Center Utrecht, 3584 CX Utrecht, The Netherlands; w.foppen@umcutrecht.nl (W.F.); n.tolboom@umcutrecht.nl (N.T.)

Medicine has evolved into a high level of specialization using the very detailed imaging of organs. This has impressively solved a multitude of acute health-related problems linked to single-organ diseases. Many diseases and pathophysiological processes, however, involve more than one organ. An organ-based approach is challenging when considering disease prevention and caring for elderly patients, or those with systemic chronic diseases or multiple co-morbidities. In addition, medical imaging provides more than a pretty picture. Much of the data are now revealed by quantitate algorithms with or without artificial intelligence. This Special Issue on “Systems Radiology and Personalized Medicine” includes reviews and original studies that show the strengths and weaknesses of structural and functional whole-body imaging for personalized medicine.

Cardiorenal syndrome is an example where physiological processes include more than one organ. Cardiac and renal functions may interact, in which the dysfunction of one organ may influence the function of the other. In the review on cardiorenal syndrome by Lin et al., imaging strategies are discussed in order to evaluate the cardiac and renal structure, function, and to characterize tissue using ultrasonography, computed tomography (CT), magnetic resonance imaging (MRI), and nuclear imaging techniques [1].

Nuclear molecular imaging techniques enable whole-body imaging and (patho)physiological processes, for example, in infectious diseases and oncology. Bloodstream infections of unknown origin, early spondylodiscitis, and vascular graft infections can be difficult to detect using standard diagnostics. Infectious diseases may be assessed by positron emission tomography (PET) CT by visualizing higher glucose metabolism using the glucose analog 2-Deoxy-2-[fluorine-18] fluoro-D-glucose (FDG) PET/CT. Common applications of FDG-PET/CT in the evaluation of infectious diseases are described by Pijl et al. [2]. In oncology, nuclear medicine is essential for staging and following up various types of cancer. In children, the most common extra-cranial solid malignancy is neuroblastoma, which can occur in the sympathetic trunk or in the adrenal medulla; about half of these children have metastatic disease at the time of diagnosis. Samim et al. reviewed the standard nuclear imaging technique (meta-[^123^I]iodobenzylguanidine ([^123^I]mIBG) whole-body scintigraphy) for the staging and response assessment of neuroblastoma, as well as new tracers and imaging techniques for neuroblastoma [3].

Vascular pathologies can occur anywhere through the body and include a wide spectrum of diseases. Imaging plays an important role in differentiating vascular pathologies which may have similar characteristics but a different etiology and thus require different treatment. For differentiation between large-vessel vasculitis and atherosclerosis, Nienhuis et al. reviewed the evidence of various imaging techniques [4]. Cerebral aneurysms may rupture, leading to morbidity and mortality. The size of the aneurysm is one of the risk factors for rupture, although other factors including morphological and hemodynamic risk factors may contribute to a better prediction of potential ruptures. Lee et al. evaluated a series of ruptured and unruptured cerebral aneurysms using cerebral angiography, and identified morphological and hemodynamic factors associated with aneurysm rupture which may be tested in larger prospective studies [5]. Peripheral artery disease and venous diseases of the legs have a huge medical and economic impact because they may eventually lead to critical limb ischemia and amputation, and deep vein thrombosis, which may cause subsequent pulmonary embolism. Imaging may be used to differentiate different patterns or causes of these diseases. Arterial calcification patterns on CT can be evaluated, for example, because these are different in patients with critical limb ischemia compared to patients without peripheral artery disease [6], and MRI flow techniques may be used to differentiate between reflux and non-reflux venous diseases [7].

Musculoskeletal diseases could severely affect patients’ mobility and well-being, of which osteoarthritis is a leading cause of disability. Multiple joints are often affected by osteoarthritis, and this may hamper interpretation of the contribution of a specific joint in patients’ quality of life, physical performance, or biochemical markers. Gielis et al. developed and tested the reproducibility of the OsteoArthritis Computed Tomography-Score in order to assess osteoarthritis burden throughout the body in large joints and the spine [8]. Abnormal new bone formation was observed in patients with diffuse idiopathic skeletal hyperostosis (DISH), especially in the spine near the anterior longitudinal ligament, resulting in an increased risk of spinal fractures. Although the exact pathophysiology is unknown, DISH is associated with obesity; Harlianto et al. reported the relationship between DISH and visceral adipose tissue based on data from over 4000 patients [9].

Artificial intelligence (AI) can have multiple applications in personalized medical imaging, ranging from personalized and faster scanning to aiding diagnosis and prognosis. The performance of AI in image reading and diagnosis is widely studied and expected to change the radiology workflow in the near future. In the past 1.5 years, COVID-19 has had a huge impact on healthcare worldwide, and numerous imaging studies using X-rays and CT for the evaluation of COVID-19 have been published in 2020–2021. Lee et al. report their findings on their evaluation of convolutional neural networks for COVID-19 screening on chest X-rays [10]. An important aspect of diagnostic imaging is the acquisition of the image during the study procedure. Adequate contrast enhancement is essential in abdominal CT studies, to ensure the accurate detection and characterization of liver lesions, for example. Patient-related factors such as height, weight, and cardiac output have an influence on the degree of contrast enhancement. De Jong et al. studied the relationship between liver enhancement and body composition assessed by an artificial intelligence algorithm. Their data and observation that lean body weight is more strongly associated with liver enhancement than total body weight and body mass index suggest that the use of an artificial intelligence body-composition-based algorithm may result in a reduction in variability in liver enhancement as well as lowering the amount of contrast media [11].

We now live in an era moving again towards a more holistic form of medicine. This movement is, in part, driven by population aging in several countries, by a multitude of novel therapeutic options, and by the impressive ability to combine systems biology, systems medicine and systems radiology data. In this Special Issue, some insight is provided to the broad range of possibilities that have already entered clinical medicine or will gain impact and create value in the near future.
